# Association between P-pulmonale and respiratory morbidity in COPD: a secondary analysis of the BLOCK-COPD trial

**DOI:** 10.1186/s12890-023-02748-2

**Published:** 2023-11-09

**Authors:** R. Chad Wade, Takudzwa Mkorombindo, Sharon X. Ling, Erika. S. Helgeson, David M. MacDonald, Krystle Pew, Helen Voelker, Vera Bittner, Ken M. Kunisaki, Matthew R. Lammi, Mark. T. Dransfield

**Affiliations:** 1 Division of Pulmonary, Allergy & Critical Care Medicine, Department of Medicine, University of Alabama at Birmingham, 1900 University BLVD, THT 422, Birmingham, AL 35294 USA; 2 Section of Pulmonary, Acute Care Service, Birmingham Veterans Affairs Medical Center, Birmingham, AL USA; 3 Division of Biostatistics, University of Minnesota, Minneapolis, MN USA; 4 Section of Pulmonary, Allergy, Critical Care, and Sleep Medicine, Department of Medicine, Minneapolis Veterans Affairs Medical Center, Minneapolis, MN USA; 5 Division of Pulmonary, Allergy, Critical Care, and Sleep, Department of Medicine, University of Minnesota, Minneapolis, MN USA; 6 Division of Cardiovascular Disease, Department of Medicine, University of Alabama at Birmingham, Birmingham, AL USA; 7 Division of Pulmonary and Critical Care Medicine, Johns Hopkins University, Baltimore, MD USA

**Keywords:** Electrocardiography, Pulmonary hypertension, Acute exacerbation, COPD

## Abstract

**Rationale:**

Pulmonary hypertension (PH) in COPD confers increased risk of exacerbations (ECOPD). Electrocardiogram (ECG) indicators of PH are prognostic both in PH and COPD. In the Beta-Blockers for the Prevention of Acute Exacerbations of COPD (BLOCK-COPD) trial, metoprolol increased risk of severe ECOPD through unclear mechanisms.

**Objective:**

We evaluated whether an ECG indicator of PH, P-pulmonale, would be associated with ECOPD and whether participants with P-pulmonale randomized to metoprolol were at higher risk of ECOPD and worsened respiratory symptoms given the potential detrimental effects of beta-blockers in PH.

**Methods:**

ECGs of 501 participants were analyzed for P-pulmonale (P wave enlargement in lead II). Cox proportional hazards models evaluated for associations between P-pulmonale and time to ECOPD (all and severe) for all participants and by treatment assignment (metoprolol vs. placebo). Linear mixed-effects models evaluated the association between treatment assignment and P-pulmonale on change in symptom scores (measured by CAT and SOBQ).

**Results:**

We identified no association between P-pulmonale and risk of any ECOPD or severe ECOPD. However, in individuals with P-pulmonale, metoprolol was associated with increased risk for ECOPD (aHR 2.92, 95% CI: 1.45–5.85). There was no association between metoprolol and ECOPD in individuals without P-pulmonale (aHR 1.01, 95% CI: 0.77–1.31). Individuals with P-pulmonale assigned to metoprolol experienced worsening symptoms (mean increase of 3.95, 95% CI: 1.32–6.58) whereas those assigned to placebo experienced a mean improvement in CAT score of -2.45 (95% CI: -0.30- -4.61).

**Conclusions:**

In individuals with P-pulmonale, metoprolol was associated with increased exacerbation risk and worsened symptoms. These findings may explain the findings observed in BLOCK-COPD.

**Supplementary Information:**

The online version contains supplementary material available at 10.1186/s12890-023-02748-2.

## Introduction

Chronic obstructive pulmonary disease (COPD) afflicts over 65 million people worldwide, resulting in 3 million deaths annually, and in the United States, direct medical costs average over ten thousand dollars annually per patient [1]. Most of the morbidity and expense related to COPD results from exacerbations (ECOPD) [2–4]. Over the past two decades, a strong independent association between COPD and cardiovascular disease (CVD) has been established, and heart-lung interactions contribute to exacerbation risk [5, 6].

Cardiovascular morbidity in COPD patients extends beyond the well-established link with ischemic heart disease. In COPD patients, pulmonary hypertension (PH) related to destruction of lung tissue and chronic hypoxia is relatively common with estimates of PH prevalence using cardiac catheterization ranging from 20 to 36%, and up to 45% with exercise [7–9]. Furthermore, PH and right heart dysfunction (RHD) are associated with risk of ECOPD, decreased survival, and reduced exercise capacity [10–13]. Despite the poor outcomes associated with PH and RHD, there are no guideline recommendations to screen for PH in COPD nor are there any available therapies. While right heart catheterization is the gold standard for diagnosis of PH, less invasive diagnostic tools, such as echocardiography and electrocardiograms (ECG), are more available and can be valuable in identifying and quantifying the severity of PH and RHD.

The Beta-Blockers for the Prevention of Acute Exacerbations of COPD (BLOCK-COPD) trial randomized participants to metropolol vs. placebo for the prevention of ECOPD. Metoprolol did not affect the overall risk of ECOPD, but increased the risk of severe exacerbations requiring hospitalization and worsened respiratory symptoms as assessed by the COPD Assessment Test (CAT) and the San Diego Shortness of Breath Questionairre (SOBQ) [14]. The effect of RHD on exacerbation outcomes in BLOCK-COPD was not explored.

Previous work revealed that P-pulmonale, an ECG indicator of RHD, is both prevalent and associated with increased mortality in PH [15–18]. In addition, the role of beta-blocker therapy in patients with PH is controversial with studies showing conflicting results and guidelines recommending against beta-blocker therapy without a strong indication [19–22]. The aim of our study was to investigate if unrecognized PH and RHD (identified by P-pulmonale) may contribute to the detrimental effects of metoprolol observed in BLOCK-COPD.

We used data from BLOCK-COPD to test the hypothesis that P-pulmonale, an indicator of RHD in PH, is associated with ECOPD and would identify individuals at further risk for increased symptoms and ECOPD when randomized to metoprolol.

## Methods

### BLOCK-COPD

BLOCK-COPD (NCT02587351) was a prospective, multi-center, placebo-controlled, double-blind, randomized trial of spirometry confirmed COPD patients aged 40–85 years [14]. Institutional Review Boards approved the study protocol at each of the 26 participating sites. After thoroughly explaining the study protocol to the patients, written informed consent was obtained from all participants. The trial assessed the effect of metoprolol (*n* = 268) or placebo (*n* = 264) on time to first exacerbation.

### Participants

All BLOCK-COPD participants had a standard 12-lead ECG performed at enrollment. We excluded participants with ECGs that were uninterpretable due to baseline artifact that obscured P waves (*n* = 31). All participants had at least moderate COPD (by post-bronchodilator spirometry) with either a history of exacerbation in the prior year or a prescription for supplemental oxygen for use at home. Patients with guideline-concordant indications for beta-blocker use, including heart failure with a reduced ejection fraction less than 40% or a history of myocardial infarction or coronary revascularization within the prior 36 months were excluded, but patients with other cardiovascular diseases were eligible.

### Exacerbations of COPD

An exacerbation of COPD was defined as an increase in or new onset of two or more of the following symptoms: cough, dyspnea, sputum production, wheezing, or chest tightness leading to treatment with either antibiotics or systemic glucocorticoids for at least three days. ECOPD severity was graded as mild (outpatient management with or without contact with a healthcare provider), moderate (resulting in emergency department visits), severe (resulting in hospitalization), or very severe (requiring mechanical ventilation). For this analysis, we evaluated ECOPD of any severity and severe ECOPD (which included both severe and very severe events).

### Procedures

Clinical assessments included demographics, CAT and SOBQ questionnaires, 12-lead electrocardiography, and pre- and post- bronchidilator spirometry [23, 24]. Symptom assessment questionnaires were collected at baseline and follow-up visits on Day 112 and Day 336. Close-out visit measurements from participants who ended the study early were analyzed as if they were measured at the next scheduled study visit.

Trained technicians collected 12-lead ECGs in a supine position at enrollment. ECGs were analyzed using electronic calipers. A group of trained physicians analyzed all ECGs, and all physicians analyzed a standard subset of ECGs to assess interobserver agreement on the presence or absence of P-pulmonale. Baseline ECGs of 501 participants were analyzed for P-pulmonale, a marker for right atrial enlargement that is evidenced by P wave enlargement > 2.5 mm in lead II [25, 26].

### Statistical analysis

Continuous values were summarized using means and standard deviations (SD) and compared between groups using ANOVA tests. Categorical variables were summarized using proportions and compared between groups using chi-square tests. Interobserver agreement was assessed using Cohen’s kappa statistic. Cox proportional hazards models were used to test the association between P-pulmonale and time to first ECOPD (any ECOPD and severe ECOPD) and to test the interaction between P-pulmonale and treatment assignment on time to first ECOPD. Adjusted models included covariates utilized in the parent BLOCK-COPD trial; age, sex, Black race, FEV_1_ percent predicted, smoking status, heart rate greater than the median value (84 bpm), number of hospitalizations for COPD during the previous year, number of exacerbations treated with glucocorticoids or antibiotics during the previous year, use of supplemental oxygen, and scores on the COPD Assessment Test and the mMRC scale (mMRC ≥ 2 vs. ≤1) and were stratified by study site. All regression models adjust for baseline values through the use of subject specific random intercepts. Kaplan-Meier plots and log-rank tests were used to evaluate the probability of remaining exacerbation-free between groups defined by presence of P-pulmonale and treatment assignment.

We evaluated the relationship between P-pulmonale and treatment assignment and change in COPD symptoms (CAT and SOBQ) using linear mixed-effects models. These models were parameterized with a three-way interaction between P-pulmonale, treatment assignment, and study visit and included a subject-specific random intercept. The mixed-effects models included a subject-specific random effect, which accounts for participants’ baseline characteristics, including sex. If the three-way interaction was not-significant (*p***-**value > 0.05), we removed the three-way interaction term and evaluated the impact of P-pulmonale on change in COPD symptoms using a two-way interaction between P-pulmonale and study visit.

All *p***-**values are two-sided and not adjusted for multiple comparisons. Statistical analyses were conducted in R version 4.2.0 (R Foundation for Statistical Computing; Vienna, Austria) (See Supplement for additional details).

##  Results

### Participants

After exclusion for ECG quality, a total of 501 of the 532 BLOCK-COPD participants were included in this analysis. The mean (SD) age was 65.1 (7.8) years, 46.5% of the participants were females and 71.3% were white. The mean post-bronchodilator FEV_1_ percent predicted was 40.6 (16.1), the mean CAT score was 20.7 (7.3), and the mean SOBQ score was 52.9 (25.8). Of this sample, 255 were assigned to metoprolol and 246 to placebo (Table 1).

**Table 1:** Patient Characteristics at Baseline by P-pulmonale. Results are presented as number (percentage) of patients, unless otherwise noted. Data were available for all participants, unless otherwise noted

	Overall (*N* = 501)	P-pulmonale		
		Absent (*N* = 438)	Present (*N* = 63)	*p*-value
Treatment Assignment				
Placebo	246 (49.1%)	210 (47.9%)	36 (57.1%)	0.218
Metoprolol	255 (50.9%)	228 (52.1%)	27 (42.9%)	
Sex				
Female	233 (46.5%)	207 (47.3%)	26 (41.3%)	0.449
Male	268 (53.5%)	231 (52.7%)	37 (58.7%)	
Age, mean ± sd	65.1 ± 7.8	65.2 ± 7.9	64.4 ± 7.0	0.468
Race				
Black	127 (25.3%)	111 (25.3%)	16 (25.4%)	0.378
Other	17 ( 3.4%)	13 ( 3.0%)	4 ( 6.3%)	
White	357 (71.3%)	314 (71.7%)	43 (68.3%)	
FEV1 after bronchodilation — % of predicted value, mean ± sd	40.6 ± 16.1	41.4 ± 15.8	35.8 ± 17.5	0.010
Heart rate, mean ± sd	84.5 ± 11.1	83.7 ± 11.0	90.3 ± 10.2	< 0.001
Current smoker	157 (31.3%)	133 (30.4%)	24 (38.1%)	0.275
Oxygen use	203 (40.5%)	172 (39.3%)	31 (49.2%)	0.172
Exacerbation history, mean ± sd				
Courses of systemic glucocorticoids or antibiotics in last 12 months	1.9 ± 1.6	1.9 ± 1.6	1.8 ± 1.6	0.572
Hospitalizations in last 12 months	0.6 ± 1.1	0.6 ± 1.1	0.7 ± 1.0	0.542
CAT score, mean ± sd	20.7 ± 7.3	20.6 ± 7.2	21.8 ± 7.5	0.228
SOBQ score^a^, mean ± sd	52.9 ± 25.8	52.6 ± 25.6	55.3 ± 27.2	0.436

*COPD *Chronic obstructive pulmonary disease, *FEV1% *Forced expiratory volume in 1-second percent predicted, *CAT *COPD Assessment Test, *SOBQ *San Diego Shortness of Breath, *P-pulmonale *P wave enlargement > 2.5 mm in lead II, CAT scores range from 0 to 40; higher scores indicate worse symptoms (minimal clinically important difference = 2 points); SOBQ scores range from 0-120; higher scores indicate worse symptoms (minimal clinically important difference = 5 points)

^a^Data available for 433 participants with P-pulmonale absent; 63 with P-pulmonale present

### Interobserver agreement

There was excellent agreement (Kappa ≥ 0.90) in P-pulmonale classification between the primary and secondary ECG observers (Supplementary Table S1).

### Associations between P-pulmonale and exacerbations

**Fig. 1:**
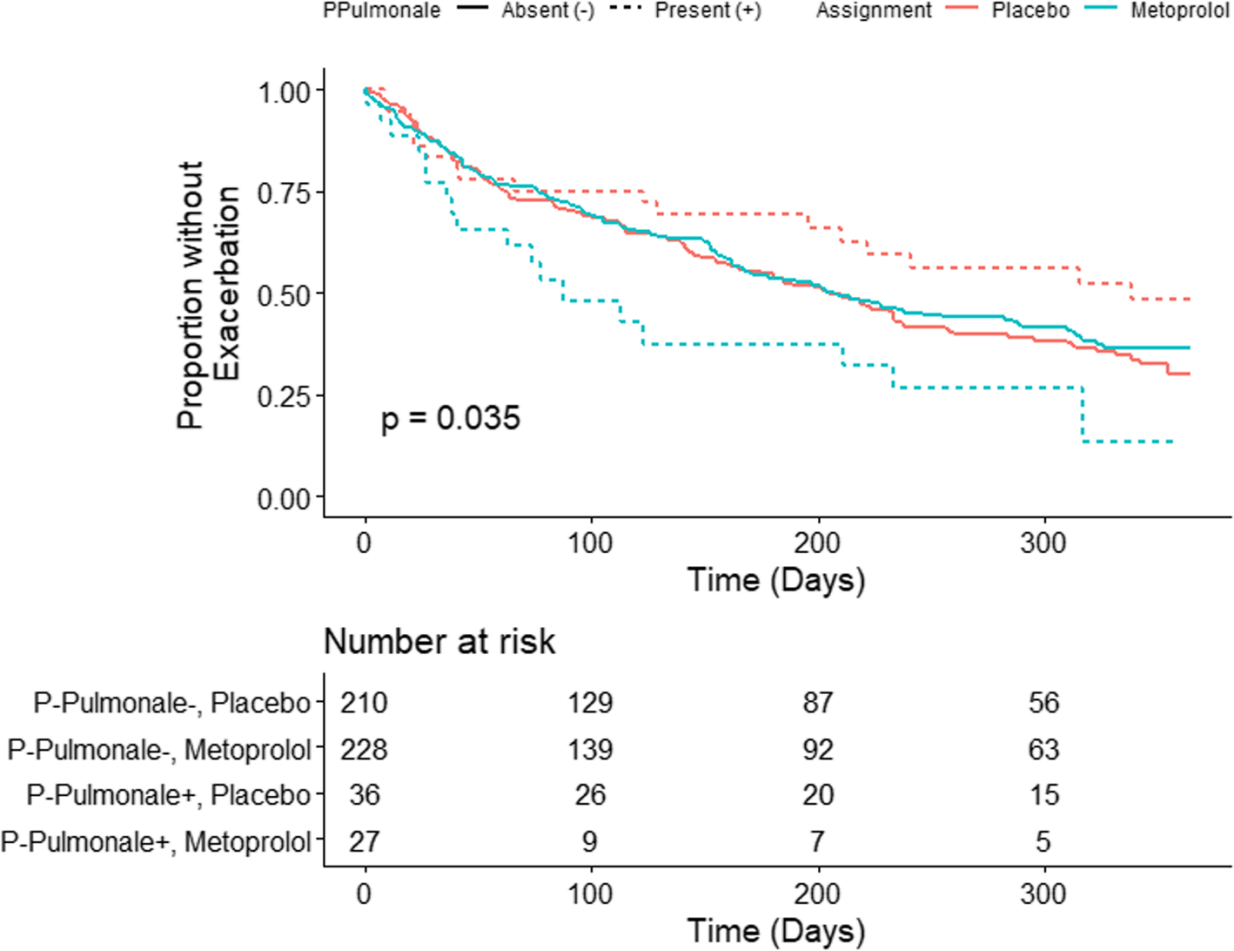
Kaplan–Meier estimate of freedom from exacerbation of COPD of any severity. Significant differences were detected between groups defined by presence of P-pulmonale and treatment assignment (logrank *p***-**value = 0.035)

P-pulmonale was observed in 63/501 participants (12.5%), with 36 (57.1%) participants with P-pulmonale in the placebo group and 27 (42.8%) in the metoprolol group (*p* = 0.22). We did not detect a statistically significant association between P-pulmonale and risk of any or severe ECOPD in the group overall (aHR 1.21, 95% CI: 0.82 to 1.78; aHR 1.30, 95% CI: 0.73 to 2.33; respectively; Supplementary Table S2). However, there was a significant interaction between treatment assignment and the presence of P-pulmonale on time to ECOPD of any severity (*p* = 0.005; Table 2; Fig. 1). Metoprolol was associated with an increased risk of ECOPD in individuals with P-pulmonale (aHR 2.92, 95% CI: 1.45 to 5.85) but not in individuals without P-pulmonale (aHR 1.01, 95% CI: 0.77 to 1.31). Metoprolol was associated with increased risk of severe ECOPD in participants with and without P-pulmonale, but we did not detect a statistically significant interaction between P-pulmonale and treatment assignment on risk of severe ECOPD (*p***-**values > 0.15).

**Table 2:** Interaction between P-pulmonale and treatment assignment (metoprolol vs. placebo) on time to exacerbations of COPD. Associations between metoprolol and time to exacerbations of COPD, stratified by P-pulmonale are presented

	Any ECOPD		Severe ECOPD	
	Unadjusted	Adjusted^a^	Unadjusted	Adjusted^a^
Interaction *p*-value	0.005	0.005	0.158	0.39
Stratified Metoprolol HR (95% CI)				
P-pulmonale -	0.93 (0.72, 1.19)	1.01 (0.77, 1.31)	1.81 (1.17, 2.81)	2.01 (1.27, 3.19)
P-pulmonale +	2.52 (1.31, 4.86)	2.92 (1.45, 5.85)	3.94 (1.48, 10.50)	3.30 (1.17, 9.28)

^a^Adjusted models included age, sex, race, FEV1% predicted, smoking status, heart rate greater than the median value, number of hospitalizations for COPD during the previous year, number of exacerbations treated with glucocorticoids or antibiotics during the previous year, use of supplemental oxygen, and scores on the COPD Assessment Test and the mMRC scale as covariates and were stratified by study site

**Fig. 2:**
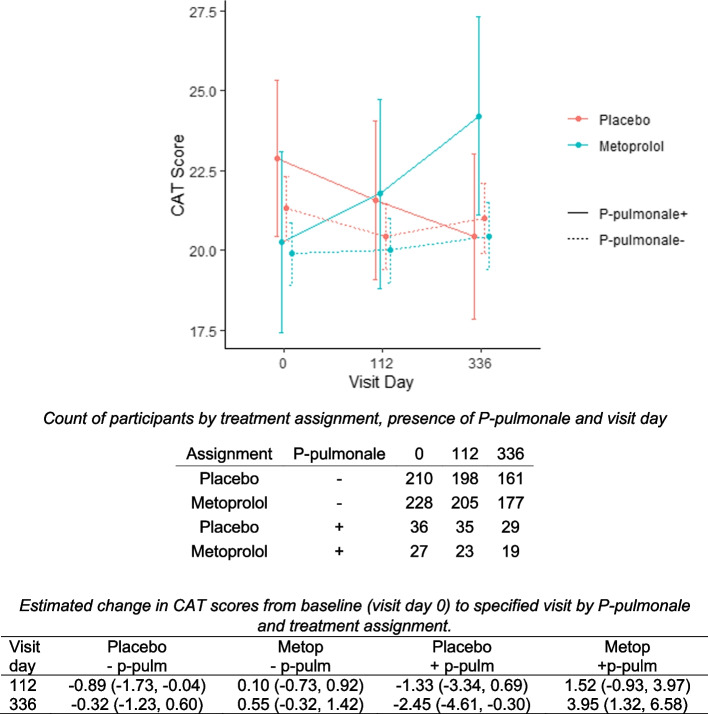
COPD Assessment Test (CAT) scores with 95% confidence intervals by assignment to metoprolol or placebo and presence of P-pulmonale. The column “P-pulmonale” indicates presence (+) or absence (-). Similarly, in the table depicting change in CAT scores, (- p-pulm) indicates absence and (+ p-pulm) indicates presence of P-pulmonale

We further evaluated the association between P-pulmonale and treatment assignment on COPD symptoms and identified an interaction between P-pulmonale and treatment assignment on change in CAT score during the study (omnibus *p***-**value for three-way interaction = 0.01; supplementary table S3; Fig. 2). Individuals with P-pulmonale who were assigned to placebo experienced a statistically significant improvement in respiratory symptoms (change in CAT score of -2.45 [95% CI: -0.30 - -4.61] from baseline to visit day 336), whereas those assigned to metoprolol experienced a stastically significant worsening of respiratory symptoms (increase in CAT of 3.95 [95% CI: 1.32–6.58) from baseline to visit day 336). Individuals without P-pulmonale had minimal change in CAT score from the beginning to the end of the study regardless of whether they were assigned to placebo (0.32 point decrease, [95% CI: -1.23-0.6]) or metoprolol (0.55 point increase, [95% CI: -0.32-1.42]).

We did not detect an interaction between P-pulmonale and treatment assignment on change in SOBQ (omnibus *p***-**values for three-way interaction > 0.2), nor did we find that P-pulmonale was associated with change in this measure (omnibus *p***-**values for two-way interaction > 0.2; Supplementary Table S4).

## Discussion

In this post-hoc analysis of the BLOCK-COPD trial, we found that patients with P-pulmonale assigned to treatment with metoprolol were at increased risk of ECOPD of any severity. In addition, metoprolol assignment was associated with worsening in respiratory symptoms in the P-pulmonale group that was both statistically and clinically significant (minimal clinically indicated differed: change in CAT > 2) with no observed change in symptoms in participants without P-pulmonale [27]. Collectively, these findings suggest that metoprolol therapy may increase respiratory symptoms and exacerbation risk in COPD patients with ECG evidence of RHD.

Our findings of increased CAT scores and exacerbation risk in those with P-pulmonale assigned to metoprolol highlight the importance identifying concomitant RHD in COPD. Overall, these findings suggest that including P-pulmonale in the risk assessment for metoprolol therapy may be helpful in this select group of COPD patients. While speculative, it is possible that metoprolol use in COPD patients with ECG signs of RHD could lead to a decrease in both inotropy and chronotropy which would reduce heart function andmanifest clinically as increased shortness of breath, a key symptom of ECOPD [21].

Previous investigators have demonstrated that the presence of PH in COPD has functional and prognostic implications and is associated with adverse COPD outcomes, increased risk for exacerbations, and more rapid lung function decline [12, 28]. Additionally, echocardiographic changes in the right atrium are associated with respiratory morbidity in COPD [11, 29, 30].

The parent BLOCK-COPD trial demonstrated that metoprolol does not reduce ECOPD risk in exacerbation-prone COPD patients with no guideline-based indication for beta-blocker therapy. In fact, metoprolol led to an increased risk of hospitalized or mechanically ventilated exacerbations as well as increased respiratory symptoms. The reasons for these findings remain unclear with subsequent post-hoc analyses of BLOCK-COPD revealing no explanatory worsening in lung function with metoprolol treatment [31]. Our current analysis identified an increased exacerbation risk and worsened symptoms with metoprolol therapy in patients with P-pulmonale and provides some potential insight into the findings from BLOCK-COPD. Our findings further highlight the impact of unrecognized and underappreciated heart-lung associations in COPD.

While there has been significant progress in understanding the heart-lung interface, much remains unknown. Structural and mechanical changes in pulmonary vasculature in COPD can increase right ventricular (RV) afterload and, in some cases, subsequently result in RV dysfunction [32–34]. Increases in RV afterload can result in RV adaptations such as augmentation of intrinsic contractility properties, increases in heart rate, and muscular hypertrophy [35]. If RV systolic function is maximized via these adaptations, and output is still inadequate, stroke volume is further increased by changes in morphology, notably RV dilation (increased preload by increasing RV end-diastolic pressure) [36, 37]. While the determinants of the transition from adaptive changes to a maladaptive state in COPD are unclear, these changes in RV morphology are associated with reduced exercise capacity and have important prognostic implications [32, 37]. Beta-blocker therapy may lead to deleterious effects in PH by reducing heart rate and intropy thus undoing some of the RV adaptations.

A better understanding of RV mechanics overall would allow for utilization of multimodal assessments of RV function and morphology and the identification of individuals at heightened risk of COPD morbidity and mortality. This would also allow for targeted investigations of PH therapies in a population enriched for individuals most likely to receive benefit.

Our study has several limitations. When compared to right heart catheterization, echocardiography, or cardiac magnetic resonance imaging (cMRI), ECG changes are insensitive to identifying RHD and PH. We focused our analysis on P-pulmonale as it has been shown to be prevalent and have a strong association with PH based on existing literature, but our lack of utilization of more sensitive measures of PH and RHD could lead to some persons with PH being incorrectly analyzed in the ‘no PH’ group which would bias our estimates towards the null. Second, only ECGs obtained at baseline were analyzed which precluded evaluation for longitudinal changes in cardiac conduction, particularly during the an exacerbation; however, this allowed us to standardize for ECGs being obtained only when patients were several weeks exacerbation free and not on study drug. Third, ECG data were analyzed retrospectively, and while observers were well qualified, blinded to patient outcomes, and utilized an electronic caliper system, there may be varying interrater assessment of our ECG indicator of PH, P-pulmonale, which may affect our results. However, in our measurement of interrater reliability, there was strong agreement in P-pulmonale classification between observers. Finally, our study differed from the parent study findings in that metoprolol was associated with an increased risk of severe and very severe exacerbations in BLOCK-COPD; whereas, in our study, we additionally found an association between metoprolol and all exacerbations in individuals with P-pulmonale. We did not find that metoprolol was associated with a greater risk of severe exacerbations in those with p-pulmonale compared to those without p-pulmonale. However, in our analysis a relatively small number of participants with P-pulmonale experienced severe/very severe exacerbations limiting our statistical power to detect associations with this outcome.

In conclusion, we found that metoprolol is associated with an increased risk of exacerbation and worsening COPD symptoms in individuals with P-pulmonale. Further investigations are needed using widely available and novel tools targeted at the impact of right heart dysfunction in COPD.

### Supplementary Information

**Additional file 1: Supplementary Table S1.** Cohen’s Kappa statistics for interrater reliability. Kappa statistic and number of ECGs read are presented for each measure. **Supplementary Table S2.** *P***-**values for test of interactions between ECG parameters and treatment assignment (metoprolol vs placebo) on time to acute exacerbations of COPD. **Supplementary Table S3.** Associations between ECG parameters and time to acute exacerbations of COPD. HR (95% CI); p-values are presented. The association was only evaluated if the interaction between the ECG parameter and treatment assignment was not statistically significant (*p***-**value>0.05, Supplementary Table S2). **Supplementary Table S4.** *P***-**values from linear mixed effects models with subject specific random intercepts evaluating the relationship between P-pulmonale and change in COPD symptoms. If the three-way interaction between visit day, treatment assignment, and presence of P-pulmonale was not significant (*p*>0.05), the three-way interaction was removed and the two-way interaction between visit day and presence of P-pulmonale was evaluated. 

## Data Availability

The datasets used and/or analysed during the current study available from the corresponding author on reasonable request.
