# Supplementary material for: Association between P-pulmonale and respiratory morbidity in COPD: a secondary analysis of the BLOCK-COPD trial
Source: BMC Pulm Med. 2023 Nov 9;23:434. doi: 10.1186/s12890-023-02748-2 (PMC10634074; doi:10.1186/s12890-023-02748-2)
Supplement: Supplementary file 1 — Additional file 1: Supplementary Table S1. Cohen’s Kappa statistics for interrater reliability. Kappa statistic and number of ECGs read are presented for each measure. Supplementary Table S2. P-values for test of interactions between ECG parameters and treatment assignment (metoprolol vs placebo) on time to acute exacerbations of COPD. Supplementary Table S3. Associations between ECG parameters and time to acute exacerbations of COPD. HR (95% CI); p-values are presented. The association was only evaluated if the interaction between the ECG parameter and treatment assignment was not statistically significant (p-value>0.05, Supplementary Table S2). Supplementary Table S4. P-values from linear mixed effects models with subject specific random intercepts evaluating the relationship between P-pulmonale and change in COPD symptoms. If the three-way interaction between visit day, treatment assignment, and presence of P-pulmonale was not significant (p>0.05), the three-way interaction was removed and the two-way interaction between visit day and presence of P-pulmonale was evaluated. [file 12890_2023_2748_MOESM1_ESM.docx]

**Supplement**

*Associations between qR pattern and acute exacerbations*

A qR pattern in V1, a marker of right ventricular strain, was present in 47/501 participants (9.4%) with 21 (44.7%) in the placebo group and 26 (55.3%) in the metoprolol group (p=0.63). There was no interaction between qR pattern in V1 and treatment assignment on the risk of any or severe AECOPD. We also did not identify an association between qR pattern in V1 and risk of AECOPD (p-values>0.4; Supplementary Tables S2-S3).

*Associations between dominant S wave and acute exacerbations*

A dominant S wave in leads V5 or V6, a marker of right ventricular hypertrophy, was observed in 53/501 (10.6%) participants, with 31 (58.5%) in the placebo group and 22 (41.5%) in the metoprolol group (p=0.19). The presence of a dominant S wave was not associated with the risk of any or severe AECOPD, and no interaction with treatment assignment was observed (p-values>0.1; Supplementary Tables S2-S3).

**Supplementary Table S1:** Cohen’s Kappa statistics for interrater reliability. Kappa statistic and number of ECGs read are presented for each measure.

|  | P-pulmonale | qR_V1 | Dominant S wave in leads V5 or V6 |
| --- | --- | --- | --- |
| Reviewer 1 vs. Reviewer 2 | 0.90 (N=50) | N/A* (N=49) | 0.69 (N=50) |
| Reviewer 1 vs. Reviewer 3 | 0.92 (N=54) | 0.55 (N=52) | 0 (N=54) |

*****Reviewer 2 reported qR_V1 absent in all ECGs evaluated. Reviewer 1 reported qR_V1 present in 5 ECGs.

**Supplementary Table S2:** P-values for test of interactions between ECG parameters and treatment assignment (metoprolol vs placebo) on time to acute exacerbations of COPD.

|  | **Any AECOPD** | | **Severe or Very Severe AECOPD** | |
| --- | --- | --- | --- | --- |
|  | Unadjusted | Adjusted^1^ | Unadjusted | Adjusted^1^ |
| P-pulmonale | 0.005^2^ | 0.005^2^ | 0.158 | 0.39 |
| qR_V1 | 0.409 | 0.441 | 0.418 | 0.528 |
| Dominant S wave in leads V5 or V6 | 0.864 | 0.613 | 0.185 | 0.428 |

1: Adjusted models included age, sex, race, FEV1 percent predicted, smoking status, heart rate greater than the median value, number of hospitalizations for COPD during the previous year, number of exacerbations treated with glucocorticoids or antibiotics during the previous year, use of supplemental oxygen, and scores on the COPD Assessment Test as covariates and were stratified by study site

2: Results are further described in Table 2

**Supplementary Table S3** Associations between ECG parameters and time to acute exacerbations of COPD. HR (95% CI); p-values are presented. The association was only evaluated if the interaction between the ECG parameter and treatment assignment was not statistically significant (p-value>0.05, Supplementary Table S2)

|  | **Any AECOPD** | | **Severe or Very Severe AECOPD** | |
| --- | --- | --- | --- | --- |
|  | Unadjusted | Adjusted^1^ | Unadjusted | Adjusted^1^ |
| P-pulmonale | N/A | N/A | 1.53 (0.92, 2.54); 0.099 | 1.3 (0.73, 2.33); 0.376 |
| qR_V1 | 0.92 (0.61, 1.39); 0.707 | 1.06 (0.69, 1.63); 0.788 | 1.13 (0.6, 2.1); 0.708 | 1.42 (0.73, 2.77); 0.305 |
| Dominant S wave in leads V5 or V6 | 1.06 (0.72, 1.54); 0.778 | 1.21 (0.79, 1.86); 0.377 | 0.82 (0.41, 1.61); 0.558 | 0.97 (0.45, 2.08); 0.935 |

1: Adjusted models included treatment assignment, age, sex, race, FEV1 percent predicted, smoking status, heart rate greater than the median value, number of hospitalizations for COPD during the previous year, number of exacerbations treated with glucocorticoids or antibiotics during the previous year, use of supplemental oxygen, and scores on the COPD Assessment Test as covariates and were stratified by study site

**Supplementary Table S4:** P-values from linear mixed effects models with subject specific random intercepts evaluating the relationship between P-pulmonale and change in COPD symptoms. If the three-way interaction between visit day, treatment assignment, and presence of P-pulmonale was not significant (p>0.05), the three-way interaction was removed and the two-way interaction between visit day and presence of P-pulmonale was evaluated.

|  | 3-way omnibus p-value | 2-way omnibus p-value |
| --- | --- | --- |
| CAT | 0.011 |  |
| SRGQ | 0.274 | 0.259 |
| SOBQ | 0.536 | 0.662 |
| 6-MWD | 0.321 | 0.219 |

**Supplement Statistics**

Statistical analyses were conducted in R version 4.2.0 (R Foundation for Statistical Computing; Vienna, Austria) (See supplement for additional details of supplement packages utilized) using versions 0.13.2, 0.7.3, 3.3.1, 1.1.29, 3.1.3, and 1.7.4.1 of the “tableone”,(1) “fsmb”, “survival”,(2) “lme4”,(3) “lmerTest”,(4) and “emmeans”(5) packages, respectively.

**References**

1. Yoshida KB, Alexander , Chipman JJ, Bohn J, McGowan LD, Barrett M, Christensen RHB. Create 'Table 1' to Describe Baseline Characteristics with or without Propensity Score Weights_. R package. version 0.13.2,. *_tableone* 2022.

2. Borgan Ø. Modeling Survival Data: Extending the Cox Model. Terry M. Therneau and Patricia M. Grambsch, Springer‐Verlag, New York, 2000. No. of pages: xiii+ 350. Price: $69.95. ISBN 0‐387‐98784‐3. Wiley Online Library; 2001.

3. Bates D, Mächler M, Bolker B, Walker S. Fitting Linear Mixed-Effects Models Using lme4. *Journal of Statistical Software* 2015; 67: 1-48.

4. Kuznetsova A, Brockhoff PB, Christensen RH. lmerTest Package: Tests in Linear Mixed Effects Models. *Journal of Statistical Software* 2017; 82: 1-26.

5. Lenth R. emmeans: Estimated Marginal Means, aka Least-Squares Means., R package version 1.4.6. ed; 2020.
